# Colouterine fistula relating to diverticulitis: a rare clinical entity

**DOI:** 10.1093/jscr/rjae035

**Published:** 2024-02-07

**Authors:** Anoosha Aslam, David J Lewis, Mayooran Veerasingham, Mohamed Z Afzal, Asar Alsaffar

**Affiliations:** Department of General Surgery, Tamworth Hospital, Dean Street, North Tamworth, NSW 2340, Australia; Department of General Surgery, Tamworth Hospital, Dean Street, North Tamworth, NSW 2340, Australia; Department of Obstetrics and Gynaecology, Tamworth Hospital, Dean Street, North Tamworth, NSW 2340, Australia; Department of General Surgery, Tamworth Hospital, Dean Street, North Tamworth, NSW 2340, Australia; Department of General Surgery, Tamworth Hospital, Dean Street, North Tamworth, NSW 2340, Australia

**Keywords:** diverticulitis, diverticular fistula, colouterine fistula

## Abstract

Colouterine fistula is a rare but recognizable complication of diverticulitis. This case illustrates the presence of a colouterine fistula in an elderly patient who had an atypical presentation for diverticulitis. She was initially treated with intravenous antibiotics for diverticulitis with a contained abscess. This gave her an opportunity to avoid surgery. However, her sepsis failed to respond to the initial treatment. Progress computerized tomography imaging demonstrated the presence of a colouterine fistula for which she required source control. Thus she underwent laparotomy, Hartmann’s procedure, and total abdominal hysterectomy with bilateral salpingo-oophorectomy. The diagnosis of colouterine fistula was confirmed intraoperatively and on histopathology. Subsequently, the patient had an uneventful recovery following the operation. This case highlights the rarity but also the veracity of this clinical entity.

## Introduction

Diverticular disease is known to be a common colonic pathology. Inflammation of diverticula can result in diverticulitis, which can then lead to complications including stricture formation, bleeding, perforation, abscess formation, and fistula formation [1].

It is reported in the literature that 4%–20% cases of diverticulitis will be complicated by fistula formation [2–4]. The most common type of fistulas associated with diverticulitis are colovesical fistulas, followed by colovaginal fistulas [5]. Colouterine fistulas are known to be rare because of the thick muscular nature of the uterus [6]. A study performed by Woods *et al*. [5] demonstrated that out of 92 patients with internal fistulas relating to diverticulitis, only three had colouterine fistulas [5].

Colouterine fistulas were first described by Jemtel [7]. Noecker [8] was the first to describe a colouterine fistula secondary to diverticulitis. Since then, there have been various case reports in the literature describing the rarity of colouterine fistulas and challenges in their management [6, 9–11].

## Case report

A 74-year-old lady, Ms XY, presented to the emergency department with 1-month history of right lower quadrant abdominal pain. She felt constipated but there were no clinical signs of obstruction. There was also no history of weight loss in the preceding months. She had reached menopause at approximately age 40, and there was no history of post-menopausal bleeding. She had two children who were born through vaginal delivery. Ms XY did not have any previous abdominal surgery and her last colonoscopy 9 years ago demonstrated multiple diverticula in the sigmoid colon. Her medical history included breast cancer, for which she had previously undergone right wide local excision and radiotherapy 3 years ago, and was in remission. Ms XY took no regular medications and she was functionally independent. On examination, she was afebrile and systemically stable. Her abdomen was clinically soft, with mild localized guarding in the right iliac fossa and left iliac fossa.

Serology demonstrated an elevated C-reactive protein (CRP) level at 123 mg/L and white cell count (WCC) of 17 × 10^9/L. Computerized tomography (CT) scanning demonstrated sigmoid diverticulitis with a 28-mm pericolic abscess. On transabdominal ultrasound, there was a bulky uterus with gas/fluid within the endometrial cavity, and endometrial thickness could not be evaluated because of the presence of gas and bowel within the pelvis.

Ms XY was treated with broad spectrum intravenous antibiotics. Her inflammatory markers were monitored, and it was noted that despite intravenous antibiotic treatment, on day 5 of admission her CRP level continued to rise to 402 mg/L and WCC increased to 31 × 10^9/L. In addition to this, a progress CT scan demonstrated ongoing sigmoid diverticulitis, and identified the presence of a fistula between the endometrial cavity and sigmoid colon. There was no drainable collection visible.

A decision was made for definitive management with a multidisciplinary operative approach. The obstetrics and gynaecology team were consulted for management of the colouterine fistula. On day 6 of admission, the general surgery team arranged for a laparotomy and Hartmann’s procedure, while the gynaecology team performed total abdominal hysterectomy with bilateral salpingo oophorectomy. A long midline laparotomy incision was made, and the sigmoid colon was mobilized followed by dissection of the sigmoid mesentery. The left ureter was identified and protected. It was noted intraoperatively that there was a large inflammatory mass of distal sigmoid colon adherent to the uterus with a fistula between the two structures. There was also a collection of pus between the sigmoid colon and the uterus. The rest of the large bowel, rectum, and small bowel was unremarkable. The sigmoid colon was divided with an NTLC 75 Ethicon stapler®. The distal resection was at the rectosigmoid junction ~5 cm from the mass and the proximal resection was at the junction of the sigmoid colon and descending colon ~10 cm from the mass. The sigmoid colon resection was followed by three pedicle hysterectomy in which the uterus was noted to be atrophic and friable. Bilateral salpingo-oophorectomy was performed after identifying both of the ureters. The cervix was excised and the vaginal vault was closed in routine fashion. After achieving haemostasis, the peritoneal cavity was thoroughly washed out. A tension-free end colostomy was fashioned on the left side of the abdomen on the preoperatively marked site. A 14 French Bellovac drain was left in the pelvis and the abdominal fascia and skin were closed as per protocol. A rectal tube was also placed.

Ms XY recovered well from the operation. On day 2, postoperatively, her pelvic drain and rectal tube were removed. She developed nausea and vomiting, consistent with ileus. A nasogastric tube was inserted with good effect. In the following days, her colostomy was active and on postoperative day 5, her nasogastric tube was removed. Her diet was gradually upgraded and her inflammatory markers were noted to be down trending (CRP was 9 mg/L and WCC was 9 × 10^9/L on postoperative day 12). She was subsequently discharged from hospital. On postoperative day 54, she was followed up in the outpatient clinic and her stoma was working well, with successful recovery from the operation.

Histopathology demonstrated perforated diverticulitis with faecal peritonitis and portion of the fistulous tract with pericolic abscess. The uterus with cervix, ovaries, and fallopian tubes showed no signs of malignancy. The presence of a colouterine fistula was confirmed with chronic suppurative inflammation of the endomyometrium.

## Discussion

The differential diagnoses of colouterine fistulas include colonic or uterine malignancy, and iatrogenic fistulas related to a foreign body or instrumentation [9]. Radiotherapy can also lead to colonic fistula formation [12].

Clinical manifestations of colouterine fistula include vaginal discharge [11]; fever and recurrent abdominal pain may not be present in the case of chronic inflammation [10]. This case report highlights a diagnostic dilemma when Ms XY presented with a 1-month history of abdominal pain and raised inflammatory markers, but there was no clinical sign of internal fistula on initial presentation. The diagnosis of colouterine fistula was subsequently established on serial imaging, and verified intraoperatively and on histopathology.

**Figure 1:**
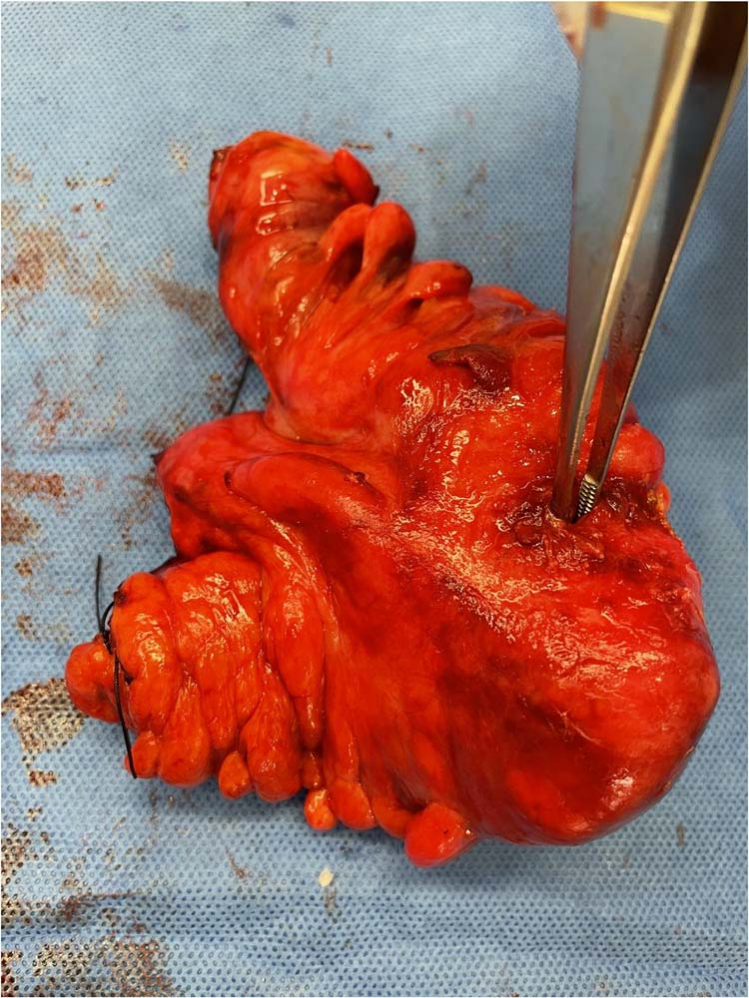
Image of the specimen containing resected sigmoid colon. A forcep is used to display the defect in the sigmoid colon wall.

**Figure 2:**
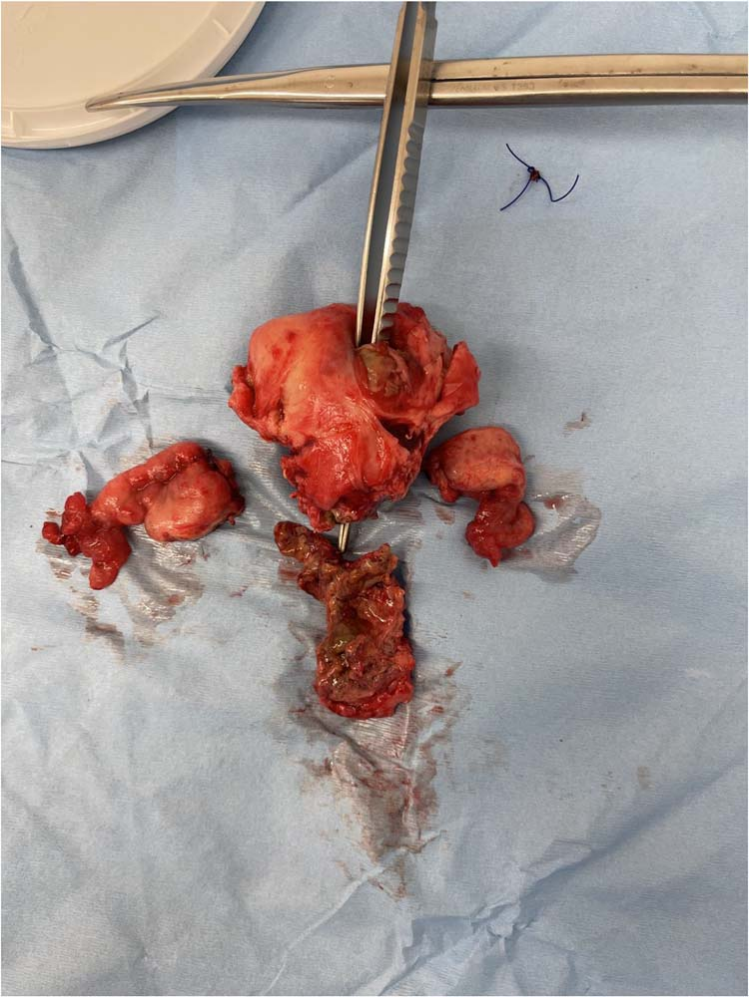
Image of the specimen containing resected uterus and bilateral ovaries. A forcep is seen probing through all layers of the uterus wall.

The most commonly used classification system for diverticulitis is the Hinchey system [13]. Originally described in 1978 [14], this was further modified in 1997, 1999, and 2005 [15–17]. The World Society for Emergency Surgery guidelines updated in 2020 suggest Hartmann’s procedure rather than primary anastomosis in critically ill patients and in selected patients with multiple comorbidities and with diverticular perforation [18]. In this case report, given the presence of purulent peritonitis intraoperatively, this would be categorized as Hinchey Stage 3 diverticulitis. Figure 1 demonstrates the point of sigmoid colon perforation, and Fig. 2 demonstrates the site of extension of the fistula into the uterus.

For definitive treatment, surgical resection of sigmoid colon is deemed necessary [6, 11]. En bloc resection of the uterus is mandated in cases of suspected malignancy; however, need for hysterectomy has not been established in benign cases [6, 19]. Another factor to consider is fertility preservation, particularly in women of child bearing age. A case report by Banky *et al.* [9] described a novel laparoscopic approach for a colouterine fistula in a 70-year-old female with severe diverticular disease, which facilitated sigmoid colectomy and preservation of the uterus. She was followed up and found to be asymptomatic at 1 year postoperatively [9]. Although the presence of colouterine fistula secondary to diverticulitis is rare, the prognosis of this is thought to be favourable [6]. Hence selection of the ideal surgical approach is vital according to the patient’s individual condition and circumstances.

In summary, acute complicated diverticulitis presents a challenge with the various pathologies involved. A multidisciplinary approach is required for these cases to provide the best possible management for patients [20]. This case report highlights the presence of a rare clinical entity, which was successfully managed with surgical resection and source control.

## References

[1] Talutis SD , Kuhnen FAH. Pathophysiology and epidemiology of diverticular disease. Clin Colon Rectal Surg 2021;34:81–5. 33642946 10.1055/s-0040-1716698 PMC7904337

[2] Menenakos E , Hahnloser D, Nassiopoulos K, et al. Laparoscopic surgery for fistulas that complicate diverticular disease. Langenbecks Arch Surg 2003;388:189–93. 12836027 10.1007/s00423-003-0392-4

[3] Maciel V , Lujan HJ, Plasencia G, et al. Diverticular disease complicated with colovesical fistula: laparoscopic versus robotic management. Int Surg 2014;99:203–10. 24833140 10.9738/INTSURG-D-13-00201.1 PMC4027901

[4] Krzak AM , Townson A, Malam Y, Mathews J. Diverticulitis complicated by colovenous fistula formation and pylephlebitis. J Surg Case Rep 2022;2022:1–3. 10.1093/jscr/rjab591 PMC8759565 35047176

[5] Woods RJ , Lavery IC, Fazio VW, et al. Internal fistulas in diverticular disease. Dis Colon Rectum 1988;31:591–6. 3402284 10.1007/BF02556792

[6] Choi PW . Colouterine fistula caused by diverticulitis of the sigmoid colon. J Korean Soc Coloproctol 2012;28:321–4. 23346512 10.3393/jksc.2012.28.6.321 PMC3548148

[7] Jemtel L . Des fistulas intestino-uterines. Arch Prov Chir 1909;1909:628–54.

[8] Noecker CB . Perforation of sigmoid and small bowel into the uterus: secondary to diverticulitis of the sigmoid. Penn Med 1929;32:496.

[9] Banky B , Marlborough F, MacLeod I, Gill TS. Single-incision laparoscopic (SIL) sigmoid colectomy and uterus-preserving repair for colo-uterine fistula secondary to severe diverticular disease: an unusual technical solution for an unusual presentation of a common disease. BMJ Case Reports 2016;2016:bcr2016214895. 10.1136/bcr-2016-214895 PMC4885370 27177935

[10] Galanis I , Floros G, Theodoropoulos C, et al. Colouterine and Jejunouterine fistula secondary to chronic diverticulitis. Case Rep Gastrointest Med 2021;2021:1–3. 10.1155/2021/5543505 PMC8032511 33868734

[11] Perez AR , Chiong-Perez ME, Arcilla CE Jr, Merin JI. Colouterine fistula: a case report of a rare complication of diverticular disease managed during the pandemic. Int J Surg Case Rep 2021;79:150–5. 33477073 10.1016/j.ijscr.2021.01.036 PMC7815980

[12] Ashburn JH , Kalady MF. Radiation-induced problems in colorectal surgery. Clin Colon Rectal Surg 2016;29:85–91. 27247532 10.1055/s-0036-1580632 PMC4882181

[13] Klarenbeek BR , de Korte N, van der Peet DL, Cuesta MA. Review of current classifications for diverticular disease and a translation into clinical practice. Int J Colorectal Dis 2012;27:207–14. 21928041 10.1007/s00384-011-1314-5 PMC3267934

[14] Hinchey EJ , Schaal PH, Richards MB. Treatment of perforated diverticular disease of the colon. Adv Surg 1978;12:85–109. 735943

[15] Sher ME , Agachan F, Bortul M, et al. Laparoscopic surgery for diverticulitis. Surg Endosc 1997;11:264–7. 9079606 10.1007/s004649900340

[16] Wasvary H , Turfah F, Kadro O, Beauregard W. Same hospitalization resection for acute diverticulitis. Am Surg 1999;65:632–5. 10399971

[17] Kaiser AM , Jiang JK, Lake JP, et al. The management of complicated diverticulitis and the role of computed tomography. Am J Gastroenterol 2005;100:910–7. 15784040 10.1111/j.1572-0241.2005.41154.x

[18] Sartelli M , Weber DG, Kluger Y, et al. 2020 update of the WSES guidelines for the management of acute colonic diverticulitis in the emergency setting. World J Emerg Surg 2020;15:32. 32381121 10.1186/s13017-020-00313-4 PMC7206757

[19] Chaikof EL , Cambria RP, Warshaw AL. Colouterine fistula secondary to diverticulitis. Dis Colon Rectum 1985;28:358–60. 3996153 10.1007/BF02560442

[20] Lambrichts DPV , Birindelli A, Tonini V, et al. The multidisciplinary management of acute complicated diverticulitis. Inflamm Intest Dis 2018;3:80–90. 30733952 10.1159/000486677 PMC6361503

